# Leveraging advanced feature extraction for improved kidney biopsy segmentation

**DOI:** 10.3389/fmed.2025.1591999

**Published:** 2025-06-18

**Authors:** Muhammad Wajeeh Us Sima, Chengliang Wang, Muhammad Arshad, Jamshed Ali Shaikh, Salem Alkhalaf, Fahad Alturise

**Affiliations:** ^1^Department of Computer Science and Technology, College of Computer Science, Chongqing University, Chongqing, China; ^2^Department of Computer Engineering, College of Computer, Qassim University, Buraydah, Saudi Arabia; ^3^Department of Cybersecurity, College of Computer, Qassim University, Buraydah, Saudi Arabia

**Keywords:** V-SAM, deep learning, adapter layer, Segment Anything Model, point-based prompt

## Abstract

Medical image segmentation faces critical challenges in renal histopathology due to the intricate morphology of glomeruli characterized by small size, fragmented structures, and low contrast against complex tissue backgrounds. While the Segment Anything Model (SAM) excels in natural image segmentation, its direct application to medical imaging underperforms due to (1) insufficient preservation of fine-grained anatomical details, (2) computational inefficiency on gigapixel whole-slide images (WSIs), and (3) poor adaptation to domain-specific features like staining variability and sparse annotations. To address these limitations, we propose V-SAM, a novel framework enhancing SAM's architecture through three key innovations: (1) a V-shaped adapter that preserves spatial hierarchies via multi-scale skip connections, recovering capillary-level details lost in SAM's aggressive downsampling; (2) lightweight adapter layers that fine-tune SAM's frozen encoder with fewer trainable parameters, optimizing it for histopathology textures while avoiding catastrophic forgetting; and (3) a dynamic point-prompt mechanism enabling sub-pixel refinement of glomerular boundaries through gradient aware localization. Evaluated on the HuBMAP Hacking the Human Vasculature and Hacking the Kidney datasets, V-SAM achieves state-of-the-art performance, surpassing 89.31%, 97.65% accuracy, 86.17%, 95.54% F1-score respectively. V-SAM sets a new paradigm for adapting foundation models to clinical workflows, with direct applications in chronic kidney disease diagnosis and biomarker discovery. This work bridges the gap between SAM's generalizability and the precision demands of medical imaging, offering a scalable solution for resource constrained healthcare environments.

## 1 Introduction

Histopathological analysis of kidney biopsies plays a pivotal role in diagnosing and monitoring chronic kidney diseases, where precise segmentation of functional tissue units (FTUs), such as glomeruli and microvasculature, is critical for quantitative assessment. While convolutional neural networks (CNNs) have advanced medical image analysis enabling breakthroughs in retinal fundus imaging, lesion detection, and MRI interpretation their reliance on large, well-annotated datasets and computational intensity remains a barrier to clinical adoption. The emergence of foundation models like the Segment Anything Model (SAM) offers transformative potential, yet its application to histopathology reveals critical gaps in handling the unique challenges of medical imaging.

Trained on over a billion natural image masks, SAM achieves remarkable zero-shot segmentation by leveraging prompt-guided inference (1). However, its performance on medical images, particularly high-resolution histopathology data, remains inconsistent. Recent benchmarks across 12 medical datasets highlight SAM's limitations in capturing fine-grained anatomical structures, with accuracy variations of up to 40% compared to specialized models (2). In renal histopathology, these shortcomings manifest in three key areas: (1) failure to distinguish texturally similar but biologically distinct regions (e.g., sclerotic vs. healthy glomeruli), (2) oversimplification of irregular microvascular boundaries, and (3) sensitivity to staining artifacts and low-contrast features prevalent in periodic acid-Schiff (PAS)-stained whole-slide images (WSIs).

Prior work in renal tissue analysis underscores these challenges. Hybrid architectures like CNN-TransXNet (3) and DLRS systems (4) have improved glomerular segmentation by combining multi-scale feature extraction with attention mechanisms. Yet, these methods require extensive task-specific tuning and lack the generalizability of foundation models. SAM's prompt driven design could bridge this gap, but its native architecture optimized for natural image semantics struggles with the hierarchical complexity of histology, where a single WSI contains structures spanning four orders of magnitude in scale (from 2μm capillaries to 200μm glomeruli).

To address these limitations, we propose a novel method that integrates SAM with a U-Net adapter to enhance segmentation performance in histological images of kidney biopsy. Our approach involves incorporating a U-Net adapter for downsampling and upsampling, along with a residual block in the image encoder to improve feature extraction. In this framework, SAM guides pixel-level segmentation, while the U-Net adapter extracts multi-scale features and refines the segmentation. The residual block enhances the capture of fine-grained details by learning residuals across layers. The detected regions are transformed into prompts and embedded in the SAM prompt encoder, which guides the segmentation process. This method aims to achieve efficient segmentation with reduced computational requirements by focusing SAM on relevant tissue areas, thereby simplifying the prediction process and eliminating the need for extensive preprocessing. Our approach introduces three key innovations:

The key contribution to this research work is given below:

- Introduce a U-Net adapter into the SAM framework, enhancing multi-scale feature extraction through skip connections, which improves segmentation accuracy and detail in complex medical images.
- A Point-Prompt Mechanism enables sub-pixel precision through adaptive refinement of point prompts via spatial gradient backpropagation, achieving precise segmentation of intricate glomerular structures while maintaining computational efficiency.
- Lightweight modules inserted into SAM's frozen encoder enable domain-specific adaptation through minimal trainable parameters (down/up-projections with ReLU). This preserves SAM's pretrained knowledge while allowing targeted feature refinement for medical textures, reducing overfitting risks on limited histopathology datasets.
- Experiments using the two datasets demonstrates the effectiveness of V-SAM. The results show significant performance improvements compared to current architectures.

The article is organized into several key sections. Section 2 provides a comprehensive review of related work in kidney segmentation, highlighting current advancements and challenges. Section 3 outlines the methodology employed in the proposed framework, detailing the approach used for improving segmentation accuracy. Section 3.2 presents a description of the datasets used in the experiments, including the data characteristics and their relevance to the study. Section 4 discusses the implementation details, covering the experimental setup, parameters, and hyperparameters that were fine-tuned for optimal performance. Section 5 showcases the results of the experiment, along with an ablation study to analyze the contribution of different components of the framework. Section 6 provides a discussion on the findings, interpreting the results in the context of existing literature. Finally, Section 7 concludes the paper with a summary of key findings and suggests directions for future research in kidney segmentation.

## 2 Related work

**Figure 1:**
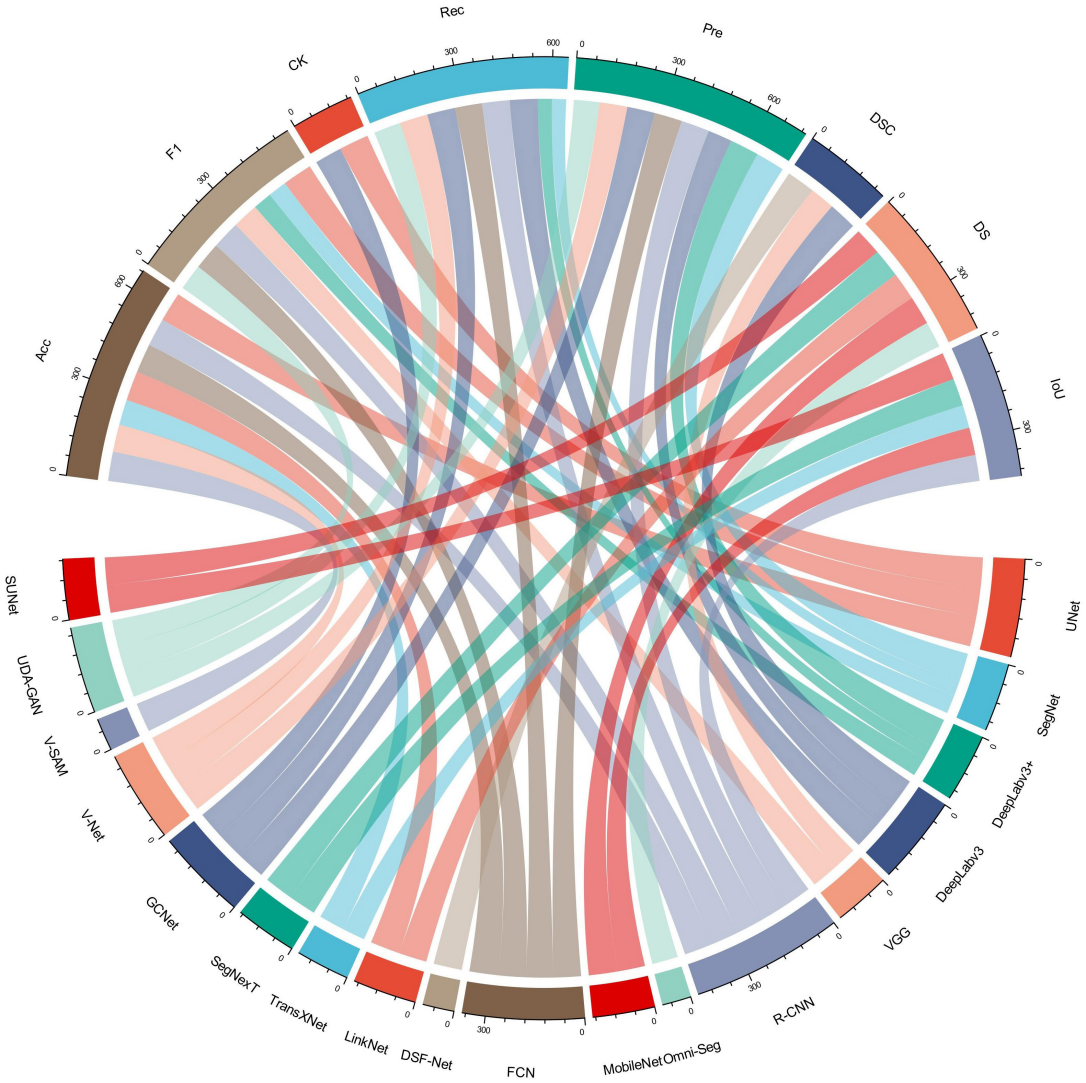
Chord plot to visualize the distributions of different models contribution for kidney glomerulus identification.

In medical image segmentation, U-Net's architecture has evolved with the integration of residual blocks to enhance gradient flow and feature extraction. Recent advancements like U-Net++ and Attention U-Net further refine segmentation accuracy. The Segment Anything Model (SAM) introduces promotable segmentation, offering interactive and flexible approaches in medical imaging. Table 1 provides an analysis of different studies in kidney segmentation methods. In kidney segmentation research, state-of-the-art models have primarily focused on accurate glomerulus identification, along with the metrics used for their validation. A chord plot (Figure 1) visualizes the contribution of these models across different validation metrics, highlighting which metrics are most commonly employed revealing key trends in evaluation practices.

**Table 1:** State-of-the-art kidney segmentation methods: purpose, approach, and performance metrics.

**References**	**WSIs**	**Glom**.	**Approach**	**Purpose**	**Performance**
(27)	47	1,245	UNet SegNet AlexNet	Using deep learning for semantic segmentation to classify glomeruli for detecting glomerulosclerosis.	**Acc:** 0.99 **F1:** 0.99 **CK:** 0.99
(28)	26	2,772	SegNet DeepLabv3+	Segmentation and classification of glomeruli for reliable estimation of the Karpinski histological score.	**Rec:** 0.47 **Pre:** 0.97 **F1:** 0.63
(29)	400	12,418	LSTM-GCNet 2D V-Net	Locate glomeruli and identify lesions like sclerosis, crescents, or none.	**CK:** 0.91 **Pre:** 0.93 **Rec:** 0.94
(30)	61	1,334	Mask-RCNN DeepLabv3	Downsampling critical glomeruli features reduces instance segmentation accuracy in high-resolution WSIs.	**DSC:** 0.95
(31)	258	24,133	VGG Unet	Developing deep learning models to automate glomeruli quantification in kidney biopsies.	**DSC:** 0.83 **F1:** 0.87 **Rec:** 0.93 **Pre:** 0.81
(32)	348	8,665	Cascade Mask R-CNN	Developing a robust model for glomeruli segmentation and classification across stains and pathologies.	**Pre:** 0.95 **Rec:** 0.92 **F1:** 0.94 **IoU:** 0.87
(33)	15	4,500	FCN-ResNet DeepLabv3	Quantification and classification of glomeruli for histopathologic assessment of renal tissue.	**Acc:** 0.97 **Pre:** 0.92 **Rec:** 0.90 F1: 0.91
(34)	146	5,459	Mask R-CNN LSTM	Examine kidney biopsies to identify glomeruli and differentiate glomerulonephritis types.	**Acc:** 0.94 **F1:** 0.94 **ROC:** 0.94
(35)	660	5,309	DS-FNet	Boundary-aware glomerulus segmentation aims to generalize across various staining methods.	**DSC:** 0.95
(36)	130	2,340	CNB-MVN-MLS	Evaluating spatial deformation augmentation for glomeruli segmentation in histopathology.	**DS:** 0.85
(37)	459	1,751	Omni-Seg	Addressing scale variations in renal WSIs with tissue-optimized multi-network segmentation.	**DS:** 0.87
(38)	20	—	LinkNet	Glomeruli detection through segmentation using neural networks.	**Acc:** 0.99 **DC:** 0.94
(39)	—	500	HistoStar-GAN UDA-GAN	Enable multi-stain transfer, normalization, and segmentation for unseen stains.	**Pre:** 0.85 **Rec:** 0.90 **F1:** 0.87
(3)	20	—	TransXNet OSRA	Capture fine details and broader context for precise segmentation.	**Acc:** 0.85 **MIoU:** 0.77
(40)	20	21,000	SegNext MobileNet	Enhancing glomeruli segmentation through cross-species pre-training on diverse kidney tissue data.	**MIoU:** 0.92 **DS:** 0.96
(41)	1,536	—	ECSA-SUNet	Variable morphology and indistinct boundaries blending with surrounding renal tissue.	**DS:** 0.92 **IoU:** 0.92
(42)	210	20,868	UniMatch_*sf*_	The resolve inter-observer variability in analyzing histopathology images.	**Pre:** 0.95 **Rec:** 0.76 **DC:** 0.81
Ours	14	13,440	**V-SAM**	Integration of FTU for segmenting blood vessels and glomeruli.	**Acc:** 0.98 **Pre:** 0.96 **Rec:** 0.95 **F1:** 0.95

WSI, Whole Slide Image; Glom, Number of Glomeruli; Pre, Precision; Rec, Recall; Acc, Accuracy; F1, F1-Score; CK, Cohen Kappa; DSC, Dice-Similarity Coefficient; DS, Dice Score; IoU, Intersection over Union; MIoU, Mean Intersection over Union; ROC, Receiver Operating Characteristic; Sens, Sensitivity; Spec, Specificity.

### 2.1 UNet

An early approach to semantic segmentation using CNNs was the Fully Convolutional Network (FCN), which struggles to recover fine details during upsampling due to spatial reduction in the downsampling process, even with transpose convolutions, resulting in coarse outputs. To address this, Ronneberger et al. (5) introduced skip connections in U-Net's encoder-decoder framework, improving segmentation accuracy. However, U-Net still faces challenges in capturing multi-scale features due to the fixed receptive field of its convolutional kernels. To improve this, Kaur et al. (6) proposed a modified U-Net model for the detection of glomeruli in images of whole-slide kidney tissue. The model improves feature extraction by adjusting filter numbers, feature map dimensions, and adding extra convolution blocks to the encoder and decoder. MLP-UNet (7) introduces a novel design avoiding traditional convolutions and self-attention mechanisms, and compares various approaches, including U-Net. For the first time, it trains the TransUNet model on the kidney WSI dataset. The TransXNet block (3), designed for glomerular segmentation, captures both fine details and broader context through a two-phase process: down-sampling with CNNs for detailed features and up-sampling with deconvolution to restore spatial resolution and enhance feature representation. UNet++ (8), an enhanced version of U-Net with a nested layer network, improves kidney biomarker predictions. DeepLabV3 (9) further enhances segmentation accuracy with atrous convolutions and atrous spatial pyramid pooling (ASPP), making it ideal for identifying four histopathological structures in acute renal tubular injury: glomeruli, necrotic tubules, healthy tubules, and tubules with casts.

### 2.2 Segment Anything Model (SAM)

The Segment Anything Model (SAM) has shown promise in medical image segmentation, leveraging its ability to generalize across diverse datasets with zero-shot learning. Its flexible prompt-based design allows for efficient segmentation of complex structures, reducing reliance on large annotated datasets. SAM's adaptability makes it a valuable tool for automated and accurate medical image analysis. SAM, originally designed for natural images, requires improvements for optimal performance on medical images (10). Leveraging SAM's ability to generate pixel-level annotations from box annotations, the authors use these SAM-generated labels to train a segmentation model. The SAM-assisted molecular-empowered learning (SAM-L) approach reduces the efforts to label the annotations of lay annotators by requiring only weak box annotations (11). A novel detector method is used to perform automatic prompted segmentation, offering a low-cost alternative to other SAM customization processes for specific tasks. det-SAM (12) features a detection head in its architecture, which provides additional domain-specific information to SAM through prompt engineering, enhancing its performance in medical segmentation tasks. DeSAM (13) introduces a decoupled SAM architecture with domain-specific prompt tuning, enhancing generalization across diverse medical imaging tasks. The framework adapts SAM's segmentation capability through anatomical-aware prompt engineering while preserving its foundational strengths. SAM2-UNet (14) integrates SAM's vision transformer encoder into a U-Net structure, demonstrating robust feature transferability for both natural and medical image segmentation. This hybrid approach achieves strong performance by combining SAM's pretrained representations with U-Net's hierarchical decoding.

Skip connections also play a vital role in preserving spatial details within encoder-decoder architectures. SAttisUNet (15) replaces direct skip pathways with an attentive module that fuses Swin Transformer features using cross-covariance attention, enhancing multi-scale context representation. The 3D framework in (16) employs convolutional blocks (CIRU, CIRC) to aggregate encoder outputs, prompt embeddings, and raw image features for improved prediction quality. Meanwhile, Yang et al. (17) propose a Deformable Squeeze-and-Attention (DSA) block that leverages deformable convolutions to adaptively refine segmentation details by learning flexible receptive fields. In contrast, our V-SAM incorporates structurally-guided skip connections that deliver multi-resolution encoder features through attention-based pathways aligned with U-shaped topology. Tailored for histological precision, these connections emphasize glomerular boundary fidelity using class-aware fusion. Unlike existing approaches, V-SAM addresses the unique challenges of glomerulus segmentation–namely, the need for fine-grained boundary preservation and prompt-driven contextual adaptation.

### 2.3 Adapter layer

The use of adapter layers in medical image segmentation has recently gained significant attention due to their ability to enhance model efficiency without the need for extensive retraining. Zhu et al. (18) introduced AdaptFormer, a transformer-based model that incorporates adapter layers, showing improved performance in medical image segmentation tasks, particularly for CT and MRI scans. Li et al. (19) also explored this concept with their dual adapter networks, which optimized segmentation accuracy for abdominal organ segmentation in CT images by combining a general-purpose backbone with task-specific adapters. Wang et al. (20) demonstrated the effectiveness of transformer-adapter networks for brain tumor segmentation in MRI scans, highlighting their capacity to extract key features with fewer parameters. Finally, Yuan et al. (21) developed dynamic adapters for multi-class segmentation tasks in lung CT scans, achieving better accuracy and efficiency in complex cases. These advancements emphasize the power of adapter layers to improve the performance of medical image segmentation models, especially when data are limited.

## 3 Methodology

**Figure 2:**
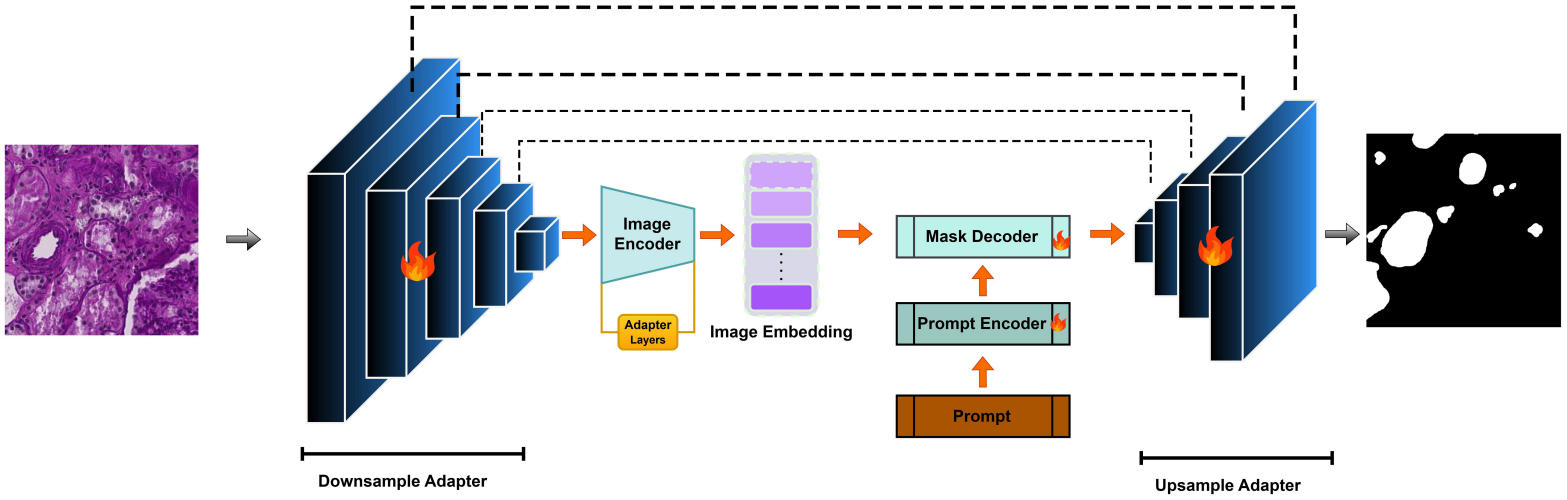
V-SAM Model: integrating a U-Net adapter into the SAM framework for enhanced multi-scale feature extraction.

Applying SAM to medical image segmentation has garnered increasing attention, with many approaches focusing on transferring knowledge from natural image domains. While SAM has demonstrated promising results in tasks involving organs or regions with large and well-defined structures, such as liver or lung segmentation, its performance declines when dealing with finer anatomical details. Tasks like blood vessel or glomerulus segmentation, which involve low-contrast boundaries, intricate morphologies, and small-scale structures, still present significant challenges highlighting the need for further architectural adaptation. In this study, we propose V-SAM, a novel architecture designed to overcome these limitations and enhance segmentation performance in the medical domain, specifically for glomerulus segmentation in kidney histology images. V-SAM integrates a promptable paradigm to improve lesion localization and more effectively capture the complex and subtle structural variations inherent in glomerular regions. It incorporates three key components: promptable information (points) for precise target area localization, a V-shaped structure to capture low-level glomerulus details, and skip connections to preserve and recover spatial information throughout the encoding-decoding process, as illustrated in Figure 2.

Segmenting FTU in kidney images presents a unique challenge due to the small size and complex shape of the glomerulus. The kidney's anatomical complexity makes it difficult for conventional segmentation models [e.g., U-Net (5), DeepLabv3+ (22), TransUNet (23), Swin-Unet (24), UNet++ (23)]. Inspired by the success of the Segment Anything Model (SAM) and its promptable segmentation paradigm (e.g., bounding boxes, points, and text), we propose to adopt this paradigm to improve the localization of glomeruli and address the unique challenges presented by kidney images. However, SAM alone struggles to achieve precise segmentation in regions with low contrast or intricate structures, as seen in kidney tissue. This challenge is particularly critical in glomerulus segmentation.

### 3.1 V-SAM framework

Following the principles of SAM, our V-SAM model uses a two-step upsampling scheme to recover the image resolution. Unlike the long-stride upsampling strategy in the original SAM, V-SAM introduces a pair of V-shaped adapters to improve segmentation accuracy. The pipeline is divided into two major phases: the downsampling encoder and the upsampling decoder. In the following sections, the image encoder is treated as part of the downsampling process, while the mask decoder is responsible for the upsampling.

#### 3.1.1 Downsampling adapter

The downsampling encoder in V-SAM serves as the hierarchical feature extractor tailored for glomerulus segmentation in gigapixel WSIs. Designed as a U-Net-inspired contraction path with V-shaped adapters, it progressively condenses spatial resolution while expanding channel capacity to capture multi-scale glomerular features from capillary-level textures to global structural contexts.

Each downsampling block implements a resolution halving operation governed by:


(1) $$\begin{array}{l} f_{i+1}=\text{Conv}\left(\text{MaxPool}\left(f_{i}\right)\right) \end{array}$$

where *f*_*i*_ and *f*_*i* + 1_ denote input/output feature maps. The MaxPool (kernel=3, stride=2) first suppresses spatial redundancy while preserving dominant activations critical for detecting glomerular capsules. The subsequent Conv layer (kernel=3, padding=1) doubles channel depth (except at *f*_4_) to expand receptive fields without sacrificing feature density – a deliberate trade-off for WSI processing where excessive downsampling erodes subtle tuft boundaries.

The five-stage feature pyramid is structured as:


(2) $$\begin{array}{l} \begin{array}{c} f_{0}\in {\mathbb{R}}^{\frac{C}{8}\times H\times W}\\ f_{1}\in {\mathbb{R}}^{\frac{C}{4}\times \frac{H}{2}\times \frac{W}{2}}\\ f_{2}\in {\mathbb{R}}^{\frac{C}{2}\times \frac{H}{4}\times \frac{W}{4}}\\ f_{3}\in {\mathbb{R}}^{C\times \frac{H}{8}\times \frac{W}{8}}\\ f_{4}\in {\mathbb{R}}^{2C\times \frac{H}{16}\times \frac{W}{16}} \end{array} \end{array}$$

With *C* = 256 matching the Channel Attention Module (CAM)'s latent dimension, this scaling ensures compatibility with SAM's pretrained weights while preventing channel explosion. The exponential channel growth (*C* → 2*C* at *f*_4_) compensates for spatial information loss, preserving texture gradients vital for distinguishing sclerotic glomeruli (collapsed capillaries) from healthy ones.

**Figure 3:**
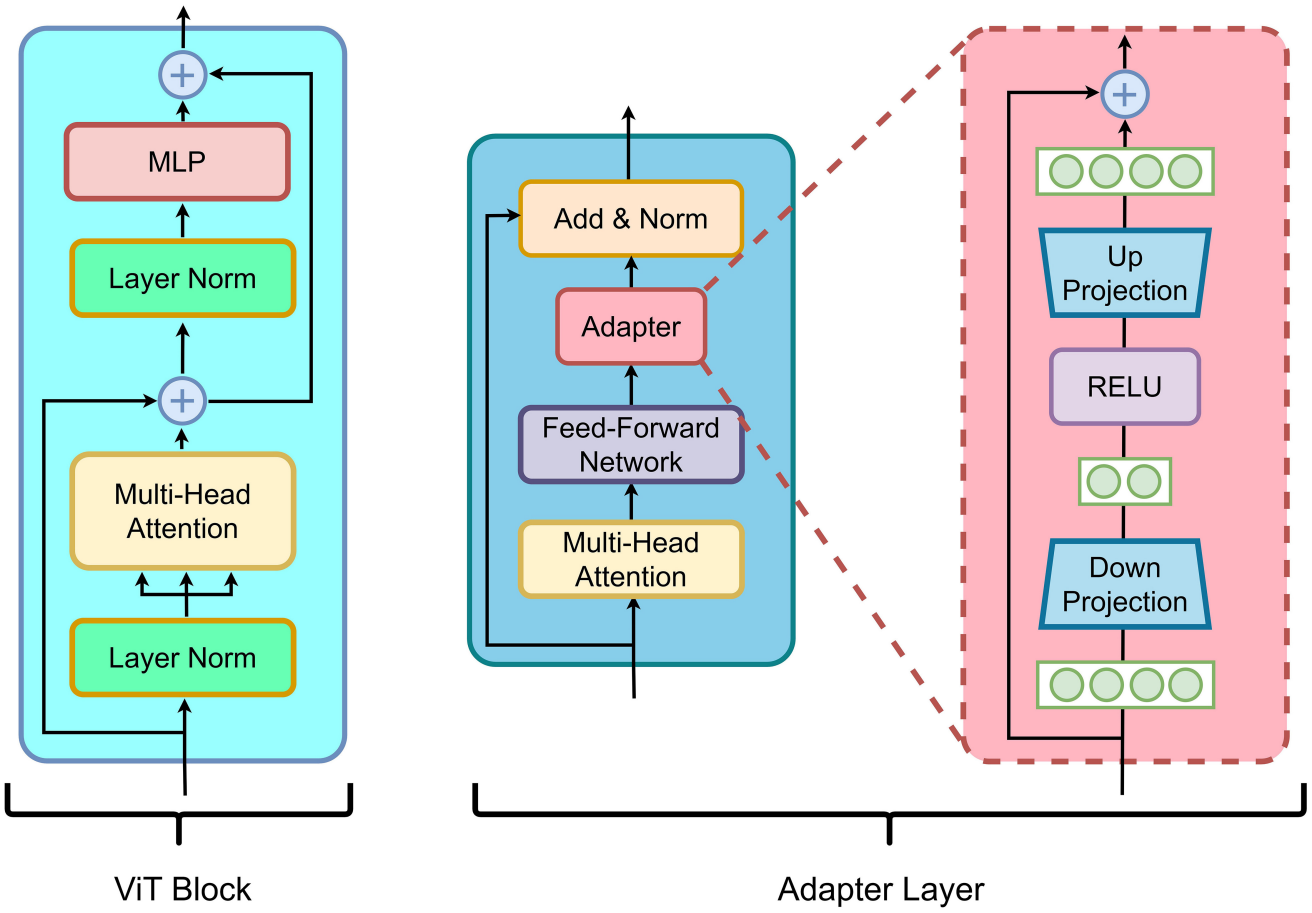
**Left** shows the ViT block capturing context, while the **right** depicts the adapter layer for enhanced multi-scale feature extraction and segmentation accuracy.

**V-SAM integration:** The deepest features *f*_4_ feed directly into SAM's image decoder as positional priors, while *f*_0_-*f*_3_ are routed via V-shaped skip connections to the upsampling decoder (Figure 2). This dual-path strategy merges SAM's semantic understanding (via *f*_4_) with U-Net's boundary precision (via skip features), crucial for resolving overlapping glomeruli in crowded WSI regions. The adapter layers (Figure 3) refine these features through lightweight down/up-projections, enabling domain adaptation without compromising SAM's generalization. For better understanding the architecture pseudo-code for V-SAM is given in Algorithm 1.

**Algorithm 1:** V-SAM Algorithm

1: **Input:** Image *I* of size 224 × 224 × 3
2: **Output:** Segmentation mask *S* of size 224 × 224 × *N*
3: let *f*_0_, *f*_1_, *f*_2_, *f*_3_, *f*_4_ be the feature maps at each downsampling stage
4: let *SAM*_*output* be the output from SAM decoder
5: let *S*_*refined* be the final refined segmentation output
6: **Step 1: Pre-process Input Image**
7: *I* ← normalize(*I*)
8: **Step 2: Downsampling Encoder**
9: **for** *i* = 0 to 3 **do**
10: *f*_*i*_ ← Encoder_Block(*I*, in_channels, out_channels)
11: *I* ← MaxPool(*I*, kernel = 3, stride = 2)
12: *I* ← Conv(*I*, kernel = 3, padding = 1)
13: **end for**
14: **Step 3: Adapter Layer**
15: *f*_4_ ← Adapter_Layer(*f*_4_)
16: **Step 4: SAM Image Encoder Processing**
17: *SAM*_*encoded* ← SAM_Encoder(*f*_4_)
18: **Step 5: SAM Decoder Processing**
19: *SAM*_*output* ← SAM_Decoder(*SAM*_*encoded*)
20: **Step 6: Upsampling Decoder**
21: **for** *i* = 3 down to 0 **do**
22: *r*_*i*_ ← Upsample_Block(*SAM*_*output, f*_*i*_)
23: *SAM*_*output* ← Upsample(*SAM*_*output*, scale_factor = 2)
24: **end for**
25: **Step 7: Final Refinement**
26: *S*_*refined* ← Final_Refinement(*SAM*_*output, f*_1_, *f*_0_)
27: **Step 8: Final Segmentation Mask Output**
28: *S* ← Final_Convolution(*S*_*refined*)
29: **Return:** *S*

#### 3.1.2 Adapter layer in image encoder

We integrate adapter layers into the image encoder of the Segment Anything Model (SAM) to adapt the pre-trained encoder for medical image segmentation tasks. Adapter layers serve as lightweight modules inserted into the encoder, introducing task-specific transformations without modifying the majority of the model's pre-trained parameters. This allows for computational efficiency by reducing the number of trainable parameters, while maintaining the encoder's generalization capability. The adapter layer is composed of two main components: a down-projection and an up-projection. The adapter layer and ViT block can be visualize in Figure 3 is The down-projection reduces the dimensionality of the feature map **h**_*l*_, and the up-projection restores the original dimension. The transformation of the input feature map **h**_*l*_ through the adapter layer is mathematically defined as:


(3) $$\begin{array}{l} {\hat{\text{h}}}_{l}={\text{h}}_{l}+{\text{W}}_{\text{up}}\cdot \sigma \left({\text{W}}_{\text{down}}\cdot {\text{h}}_{l}+{\text{b}}_{\text{down}}\right)+{\text{b}}_{\text{up}} \end{array}$$

Where *h*_*l*_ represents the output from the previous layer, *W*_down_ and *W*_up_ are the weight matrices for the down-projection and up-projection layers, respectively. The activation function is denoted by σ, and *b*_down_ and *b*_up_ correspond to the bias terms for the down-projection and up-projection layers.

Here, **h**_*l*_ is the input feature map from the encoder layer, The term σ is a non-linear activation function ReLU. The output ${\hat{\text{h}}}_{l}$ represents the adapted feature map, which is used for downstream segmentation tasks. For fine-tuning, the parameters of the adapter layers, i.e., **W**_down_, **W**_up_, **b**_down_, **b**_up_, are trained, while the other encoder parameters are kept frozen. The fine-tuning objective is to minimize the segmentation loss $L_{\text{seg}}$, which measures the difference between the model's predicted segmentation $S\left({\hat{\text{h}}}_{l}\right)$ and the ground truth *y*_gt_:


(4) $$\begin{array}{l} L_{\text{seg}}=L_{\text{seg}}\left(S\left({\hat{\text{h}}}_{l}\right),y_{\text{gt}}\right) \end{array}$$

Where $S\left({\hat{\text{h}}}_{l}\right)$ is the segmentation output generated from the adapted feature map, and *y*_gt_ is the ground truth label. The parameter efficiency of the adapter layer is given by:


(5) $$\begin{array}{l} P_{\text{adapter}}=\left(d\times r\right)+\left(r\times d\right)+r+d \end{array}$$

This results in significantly fewer trainable parameters compared to fine-tuning the entire encoder. The computational complexity of the adapter layer is:


(6) $$\begin{array}{l} O\left(d\times r\right)+O\left(r\times d\right)=O\left(2\times d\times r\right) \end{array}$$

Since *r* ≪ *d*, the computational cost is minimal. Furthermore, by training only the adapter layer parameters, we reduce the risk of overfitting, which is particularly beneficial when working with smaller medical datasets. This efficiency enables faster convergence and makes the approach suitable for resource-constrained environments.

#### 3.1.3 Prompt (point-based)

Unlike the default static point prompt strategy in SAM, our method introduces an enhanced point-based prompt mechanism tailored for multi-class medical segmentation and improved spatial precision. The proposed approach provides sparse but highly informative guidance by integrating three key innovations:


(7) $$\begin{array}{l} {\text{E}}_{p}=\overset{K}{\underset{k=1}{\sum }}\text{MLP}\left(\text{PE}\left(x_{k},y_{k}\right)⊕\phi \left(c_{k}\right)\right) \end{array}$$

Here, $\text{PE}:{\mathbb{R}}^{2}\to {\mathbb{R}}^{d_{p}}$ generates positional embeddings using sinusoidal encoding, while $\phi \left(c_{k}\right)\in {\mathbb{R}}^{d_{c}}$ maps discrete class identifiers (e.g., glomeruli, vessels, uncertain regions) to learned embeddings. The concatenation ⊕ allows the prompt to encode both spatial and semantic (class-level) information. This formulation supports multi-class segmentation directly within the prompting mechanism, a functional extension beyond SAM's binary (foreground/background) prompt treatment.

To integrate the point prompts with the model's features, we introduce a constrained cross-attention mechanism between encoded prompts and encoder outputs *f*_*i*_:


(8) $$\begin{array}{l} {\text{A}}_{m}=\sigma \left(\frac{\text{Q}{\text{K}}^{⊤}}{\sqrt{d}}\right)\text{V},\;\{\begin{array}{c} \text{Q}={\text{W}}_{q}{\text{E}}_{p}\\ \text{K}={\text{W}}_{k}f_{i}\\ \text{V}={\text{W}}_{v}f_{i} \end{array} \end{array}$$

where σ denotes row-wise softmax, and **W**_{*q, k, v*}_ are linear projection matrices. This constrained attention formulation allows the model to selectively focus on relevant spatial regions while significantly reducing memory usage compared to dense attention used in SAM. Finally, we introduce a gradient-based refinement step during training, which adapts the prompt coordinates by backpropagating segmentation loss gradients with respect to spatial positions:


(9) $$\begin{array}{l} \Delta x_{k}=-\eta \frac{\partial L_{\text{seg}}}{\partial x_{k}}=-\eta \underset{i,j}{\sum }\frac{\partial L_{\text{seg}}}{\partial {\text{A}}_{m}^{\left(i,j\right)}}\frac{\partial {\text{A}}_{m}^{\left(i,j\right)}}{\partial x_{k}} \end{array}$$

where η is the learning rate. This approach allows the prompt positions to be optimized during training for finer localization accuracy, resulting in sub-pixel alignment without any additional inference cost.

#### 3.1.4 Upsampling adapter

The V-SAM decoder extends SAM's mask generation through a hierarchical refinement process that progressively recovers spatial resolution using multi-scale encoder features. Given the initial mask source $S_{\text{src}}\in {\mathbb{R}}^{C\times \frac{H}{16}\times \frac{W}{16}}$ and transformed mask tokens $M_{t}^{\prime }$ from SAM's core processing, our architecture introduces three sequential upsampling stages:


(10) $$\begin{array}{l} \begin{array}{c} r_{3}={\text{Upsample}}_{4}\left(S_{\text{src}}⊕f_{4}\right)\\ r_{2}={\text{Upsample}}_{3}\left(r_{3}⊕f_{3}\right)\\ r_{1}={\text{Upsample}}_{2}\left(r_{2}⊕f_{2}\right) \end{array} \end{array}$$

where ⊕ denotes channel-wise concatenation with skip connections from the downsampling encoder's feature pyramid. Each Upsample_*i*_ operation consists of 2 × bilinear upscaling, 3 × 3 convolution with stride 1, ReLU activation and feature fusion with encoder skip connection *f*_*i* + 1_.

The final resolution enhancement to $\frac{H}{2}\times \frac{W}{2}$ is achieved through an additional refinement layer:


(11) $$\begin{array}{l} L={\text{Upsample}}_{1}\left(r_{1}⊕f_{1}⊕f_{0}\right) \end{array}$$

where $f_{0}\in {\mathbb{R}}^{\frac{C}{8}\times H\times W}$ provides high-frequency spatial details from the initial encoder stage. This contrasts with SAM's original approach that directly upscales low-resolution logits ($\frac{H}{16}\to H$) through a single 4 × bilinear interpolation, resulting in lost boundary details critical for glomerulus segmentation.

The complete upsampling chain reduces effective stride from 16 × to 2 × through:


(12) $$\begin{array}{l} \text{Total Upscale Factor}=\overset{4}{\underset{i=1}{\prod }}2=16\to \frac{1}{2} \end{array}$$

preserving morphological details through four gradual refinement stages rather than SAM's single coarse upsampling. This proves essential for maintaining capsule boundary continuity and resolving sub-glomerular structures in high-magnification WSIs.

### 3.2 Datasets

In this following section we will briefly explain the characteristics of two datasets used in our experiments.

#### 3.2.1 HuBMAP - Hacking the Human Vasculature (HubMAP-1)

**Figure 4:**
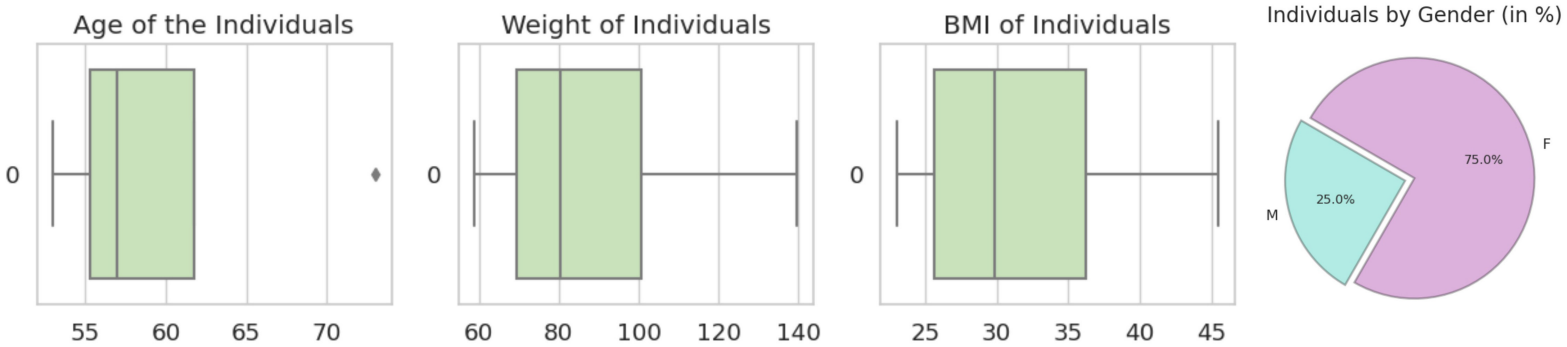
From left to right: the first image shows the age distribution among patients, the second displays weight distribution, the third illustrates body mass index (BMI) distribution, and the last image presents the dataset's gender distribution (male vs. female) in percentages.

**Figure 5:**
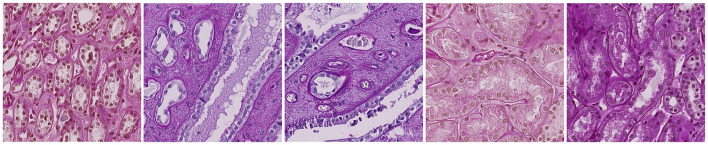
Some examples of image tiles taken from HubMAP-1.

The *HubMAP-1* dataset, originally part of the *HuBMAP-Hacking the Human Vasculature* challenge on Kaggle, is now publicly available for open use. This dataset aims to support mapping the human vascular system, focusing on functional tissue units like glomeruli and blood vessels. It includes 2D periodic acid-Schiff (PAS)-stained tiles (512 × 512 pixels) from 14 whole slide images (WSIs) of human kidney histology, selected specifically for microvascular structure segmentation. The demographic data from the data set are shown in Figure 4, with expert-verified annotations for accuracy. Additional tile information provided by dataset distributor is provided in Table 2, and training set examples are displayed in Figure 5.

**Table 2:** Attributes and metadata of the HubMAP-1 dataset.

**Attributes**	**Description**
source_wsi	The tile is extracted from which WSI
ID	ID number of the tile
i | j	The location of the upper corner from which the WSI is cropped
Age, sex, race, height, weight, BMI	Demographics of the tissue donors

#### 3.2.2 HuBMAP—hacking the kidney (HubMAP-2)

**Figure 6:**
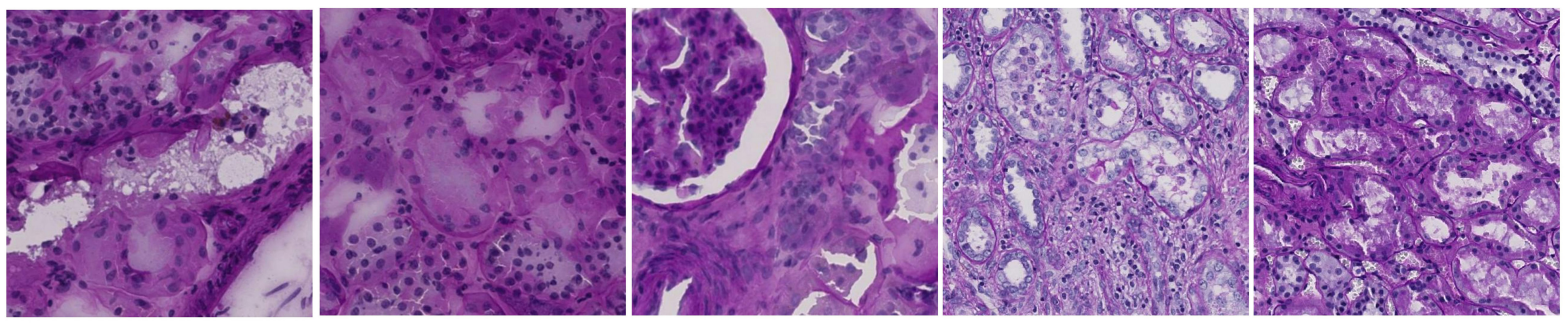
Some examples of image tiles taken from HubMAP-2.

**Figure 7:**
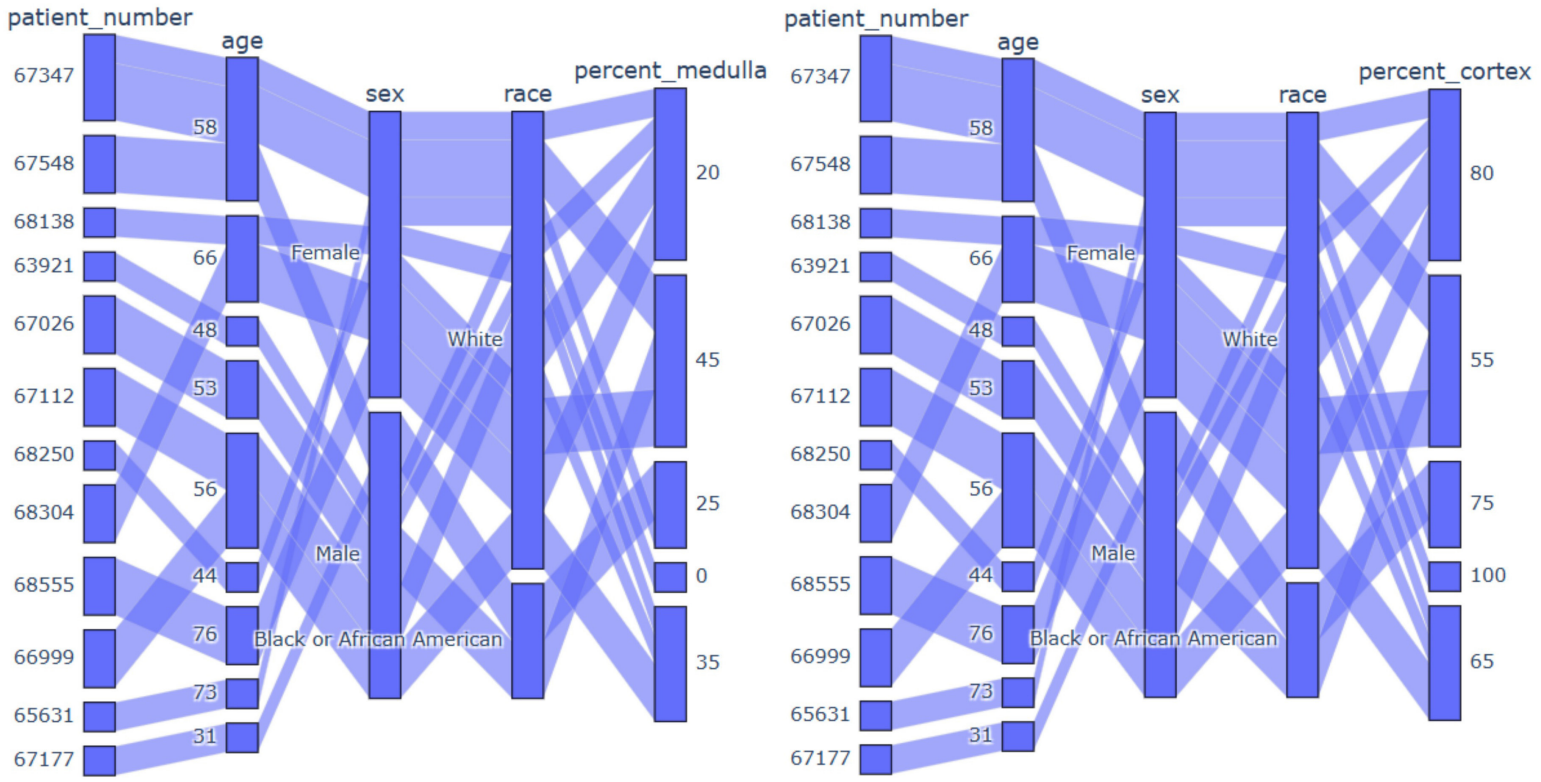
Demographic distribution by tissue composition. **Left**: age, sex, and race distribution in relation to medulla percentage. **Right**: age, sex, and race distribution in relation to cortex percentage for HubMAP-2 dataset.

The National Institutes of Health (NIH) funded the *HubMAP-2* project, which is now publicly available on Kaggle. *HuBMAP—Hacking the Kidney*, project aimed to create a detailed map of the human vascular system, with a particular focus on functional tissue units known as glomeruli. The dataset includes 11 fresh frozen and 9 Formalin Fixed Paraffin Embedded (FFPE) PAS kidney images. The TIFF images, with dimensions exceeding 19,780 × 26,840 (ranging from 182.65 MB to 4.87 GB), feature annotations of glomeruli in two formats: RLE-encoded and unencoded (JSON). Additional metadata is provided in Table 3, and sample image tiles from the training set are shown in Figure 6. Demographics for HubMAP-2 is given in Figure 7.

**Table 3:** Attributes and metadata of the HubMAP-2 dataset.

**Attributes**	**Description**
width/height pixels	Image resolution details
patient number	Tissue donor patient identifier
Race, Ethnicity, Sex, Age	Demographics of the tissue donors

## 4 Implementation

**Figure 8:**
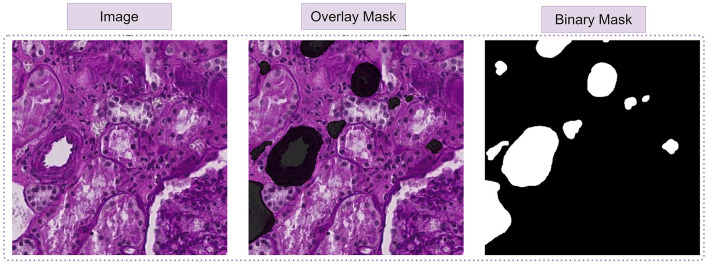
From left to right: in first image, display the dataset image. In the second image, depict polygons drawn on the corresponding image. In the third image, exhibit the binary mask of that image.

**Figure 9:**
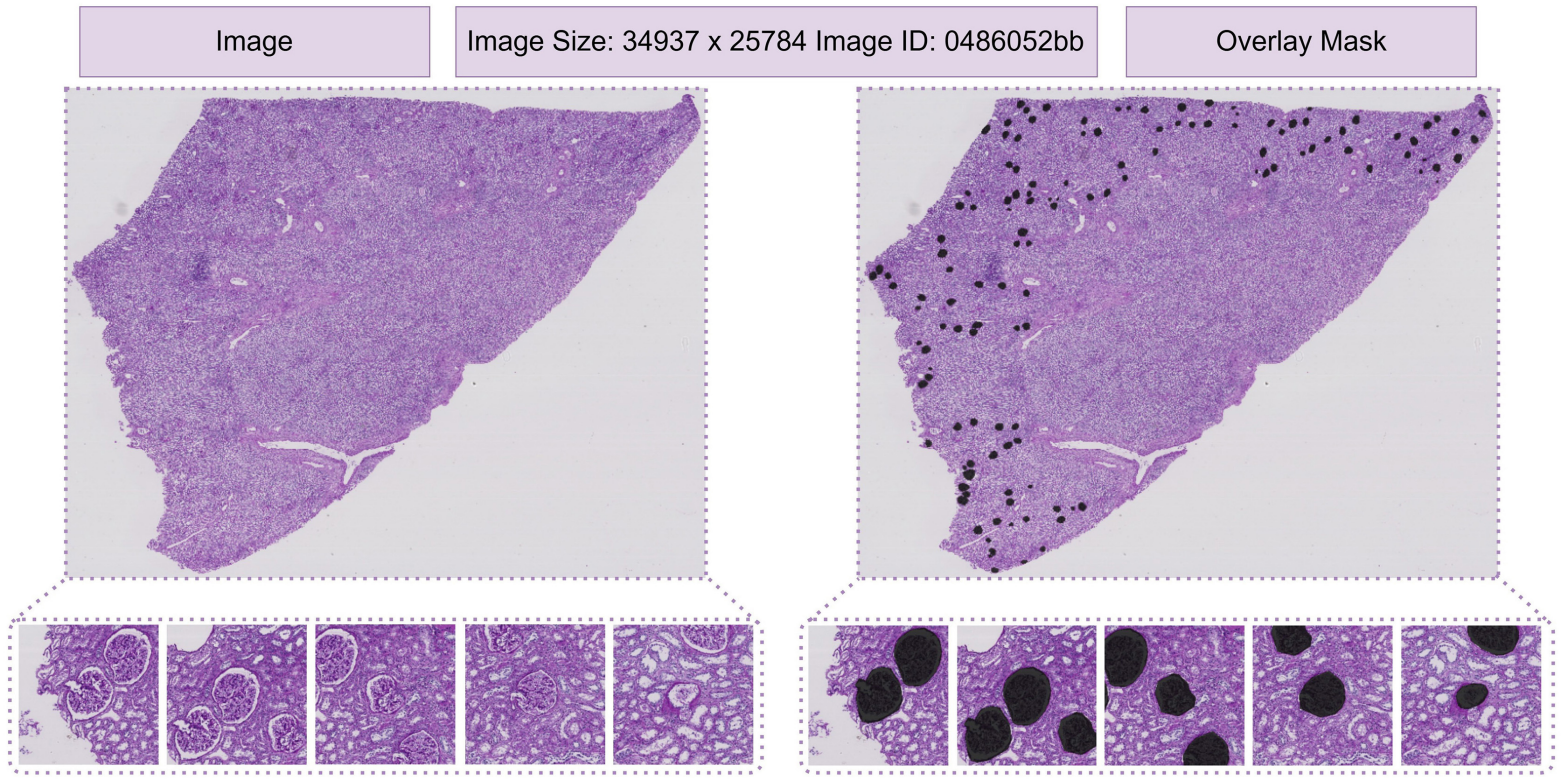
From left to right: in first image, display the dataset image. In the second image, depict polygons drawn on the corresponding image.

The experiments were carried out on a single NVIDIA RTX 3090 GPU using the PyTorch framework. To prevent overfitting, we applied data augmentations and other image slide pre-processing techniques, detailed in Table 4. Pre-trained weights from SAM on natural images were utilized to accelerate convergence and enhance training stability. During training, we froze the parameters of SAM's prompt encoder for the same purpose. While SAM was originally designed for larger input sizes, such as 1024x1024, we adapt it to handle smaller inputs like 224x224, similar to how TransUNet adapts Transformer-based architectures for reduced image sizes. In TransUNet (25), the model effectively processes smaller input sizes by leveraging a Transformer encoder-decoder structure that captures long-range dependencies while preserving fine-grained details through skip connections. For our implementation, the input resolution is set to 224x224, with a batch size of 8, enabling efficient processing while maintaining segmentation accuracy. The model was trained end-to-end using the Adam optimizer (26), with an initial learning rate of 0.0001 to speed up convergence. The parameters setting is listed in Table 5. The masking for HubMAP-1 and HubMAP-2 is given in Figures 8, 9 respectively.

**Table 4:** Augmentation parameters for the experiment.

**Attributes**	**Description**
CLAHE	True
Diagonal shift	True
Horizontal shift	True
Hue saturation	True
IAAPiecewiseAffine	0.3
Optical distortion	0.3
Random brightness	True
Random contrast	True
Vertical shift	True

**Table 5:** Parameters settings for the experiment.

**Parameters**	**Setting**
Batch size	8
Epoch	100
Initial learning rate	1 × 10^−4^
Loss function	*L*_BCE_ + *L*_Dice_
Optimizer	Adam
Patch size	224 × 224

The loss function used is a combination of binary cross-entropy and the Dice coefficient. It is formulated as:


(13) $$\begin{array}{l} L\left(Y,\hat{Y}\right)=-\frac{1}{N}\overset{N}{\underset{b=1}{\sum }}\left(\frac{1}{2}Y_{b}\log{\hat{Y}}_{b}+\frac{2Y_{b}{\hat{Y}}_{b}}{Y_{b}+{\hat{Y}}_{b}}\right) \end{array}$$

Where Ŷ_*b*_ and *Y*_*b*_ denote the predicted probabilities and the ground truth values (both flattened) for the *b*-th image in the batch, respectively, and *N* represents the batch size.

## 5 Results

### 5.1 Results on HubMAP-1

The results demonstrate that V-SAM achieves exceptional performance on the HubMAP-1 dataset, with an Accuracy of 89.31%, a Recall of 87.29%, and F1-Score of 86.17%. These scores consistently surpass a range of existing models, including both convolution-based and transformer-based architectures. Compared to UNet++ (ResNet-50), V-SAM delivers a ↑3.69% gain in Accuracy, ↑3.77% in Recall, and ↑3.26% in F1-Score. Against nnUNet, which uses a standard UNet backbone, V-SAM improves Accuracy by ↑3.12%, Recall by ↑3.02%, and F1-Score by ↑2.18%. When compared with DRA-Net (ResNet-34), V-SAM achieves ↑2.05% higher Accuracy, ↑1.66% higher Recall, and ↑1.48% higher F1-Score. Notably, although DET-SAM employs the same ViT-B backbone, V-SAM significantly outperforms it by ↑4.52% in Accuracy, ↑3.54% in Recall, and ↑5.03% in F1-Score.

**Figure 10:**
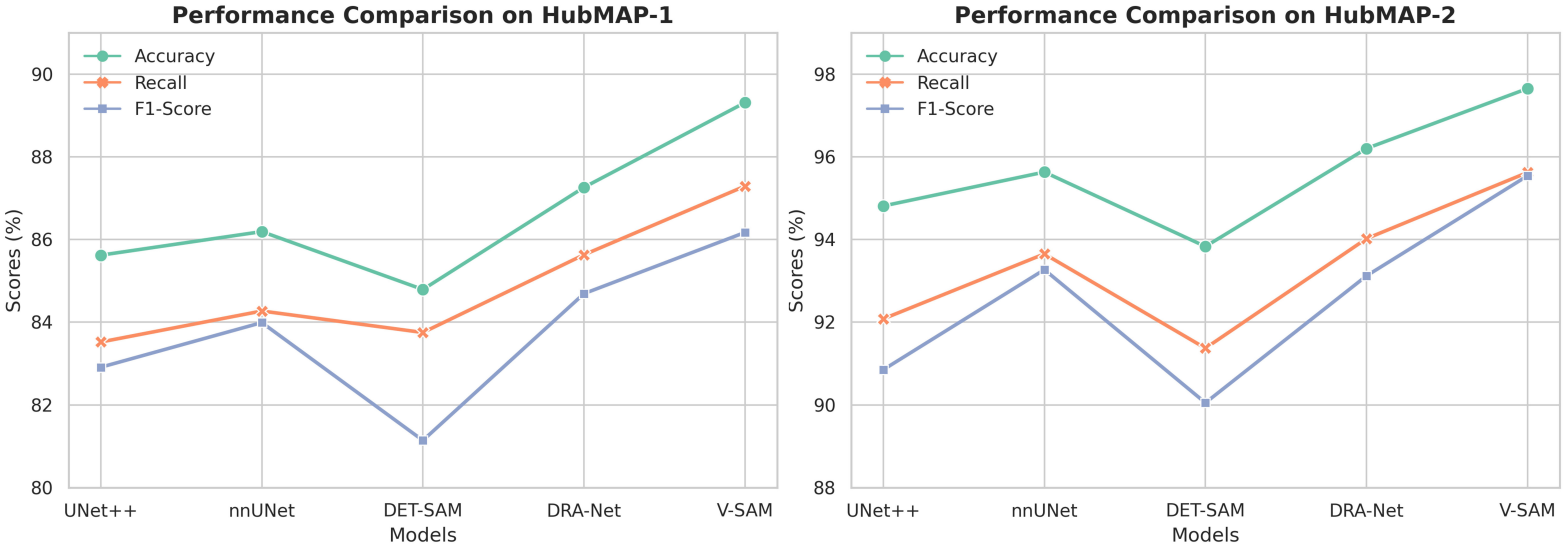
The trend of performance improvement across different models on both datasets. From left to right, the figure shows the incremental growth in Accuracy, Recall, and F1-Score for HubMAP-1 and HubMAP-2, respectively.

These consistent improvements highlight key limitations in convolution-based models like ResNet, which struggle with modeling long-range dependencies and preserving fine-grained spatial details due to their localized receptive fields. While DET-SAM shares the ViT-B transformer backbone with V-SAM, its performance is hindered by suboptimal prompting and lack of targeted skip connection design. In contrast, V-SAM incorporates a structurally guided attention mechanism, topology-aware prompting, and class-sensitive skip connections, which collectively contribute to robust segmentation of fine structures such as glomeruli. Full quantitative results are summarized in Table 6, and the corresponding performance trend is visualized in Figure 10.

**Table 6:** Comparison of V-SAM with different models for HubMAP-1.

**Model**	**Backbone**	**Accuracy (%)**	**Recall (%)**	**F1-score (%)**
UNet++ (43)	ResNet-50	85.62	83.52	82.91
nnUNet (44)	UNet	86.19	84.27	83.99
DRA-Net (45)	ResNet-34	87.26	85.63	84.69
DET-SAM (12)	ViT-B	84.79	83.75	81.14
V-SAM	ViT-B	**89.31**	**87.29**	**86.17**

The bold values showing best results.

Furthermore, V-SAM's performance is compared with recent state-of-the-art (SOTA) models on the HubMAP-1 dataset. V-SAM achieves an Accuracy of 89.31% and an F1-Score of 86.17%, surpassing UniMatch_sf (MiT-B1 backbone) by 4.77% and Omni-Seg (ReS-UNet backbone) by 3.87% in F1-Score, while achieving a remarkable 28.17% improvement over FastAI U-Net (ResNet-50 backbone), which only achieves an F1-Score of 58.00%. This performance gap underscores the effectiveness of V-SAM's transformer-based architecture, which excels at capturing global context and long-range dependencies, unlike convolutional backbones such as ResNet or ReS-UNet. Although UniMatch_sf and Omni-Seg demonstrate strong performance, their reliance on convolutional architectures limits their ability to model complex structures and global context effectively. In contrast, V-SAM's advanced fine-tuning techniques and adaptability to complex structures in medical images contribute to its superior performance, making it a robust and reliable model for medical image segmentation tasks. The comparison with SOTA models is provided in Table 7.

**Table 7:** Comparison of V-SAM with SOTA for HubMAP-1.

**Model**	**Publication/ Year**	**Backbone**	**Accuracy (%)**	**F1-Score (%)**
FastAI U-Net (46)	EESS-23	ResNet-50	—	58.00
UniMatch_sf (42)	MIDL-24	MiT-B1	—	81.40
Omni-Seg (47)	SPIE.MI-23	ReS-UNet	—	82.30
V-SAM	—	ViT-B	**89.31**	**86.17**

The bold values showing best results.

The segmentation results reveal a clear distinction in the performance of different models. UNet++ captures most major vascular structures but struggles with fine details and boundary precision, often displaying over-segmentation in certain regions, as highlighted in the red boxes. DRA-Net demonstrates improved continuity and connected component detection but still faces challenges with boundary refinement and misses smaller structural elements. Det-SAM further enhances boundary precision and reduces over-segmentation; however, it under-segments certain areas, leading to the loss of finer vessel branches. In contrast, the V-SAM achieves the most accurate segmentation, closely replicating the ground truth. It effectively captures both large and small structures with clean, precise boundaries and maintains detail even in complex regions. This superior performance is likely attributed to advanced spatial, channel, and temporal attention mechanisms that enhance feature extraction and boundary refinement. Additionally, V-SAM appears to generalize better, handling variations in noise and texture more effectively than the other models. Overall, V-SAM strikes the optimal balance between accuracy and detail preservation, making it the best-performing model in this evaluation.

### 5.2 Results on HubMAP-2

The performance of V-SAM on the HubMAP-2 dataset is thoroughly evaluated against several competitive models in Table 8, using Accuracy, Recall, and F1-Score as key metrics. V-SAM achieves the highest results across all three: 97.65% Accuracy, 95.62% Recall, and 95.54% F1-Score. Compared to UNet++ (ResNet-50), V-SAM shows significant gains of ↑2.84% in Accuracy, ↑3.54% in Recall, and ↑4.69% in F1-Score, indicating its superior ability to capture fine-grained features and global structure. Against nnUNet, which uses a self-configuring UNet-based pipeline, V-SAM improves performance by ↑2.12% in Accuracy, ↑1.97% in Recall, and ↑2.27% in F1-Score. Although nnUNet performs strongly due to its robust configuration heuristics, it still relies on standard convolutional designs, which limits its ability to model long-range dependencies.

**Table 8:** Comparison of V-SAM with different models for HubMAP-2.

**Model**	**Backbone**	**Accuracy (%)**	**Recall (%)**	**F1-Score (%)**
UNet++ (43)	ResNet-50	94.81	92.08	90.85
nnUNet (44)	UNet	95.63	93.65	93.27
DRA-Net (45)	ResNet-34	96.20	94.02	93.12
DET-SAM (12)	ViT-B	93.83	91.37	90.05
V-SAM	ViT-B	**97.65**	**95.62**	**95.54**

The bold values showing best results.

In comparison to DRA-Net (ResNet-34), which introduces attention mechanisms and dynamic refinement, V-SAM achieves ↑1.45% higher Accuracy, ↑1.60% better Recall, and ↑2.42% improved F1-Score, showcasing its more effective context modeling and class-sensitive segmentation. While DET-SAM uses the same ViT-B backbone as V-SAM, its performance is notably lower across all metrics, with V-SAM achieving ↑3.82% better Accuracy, ↑4.25% higher Recall, and ↑5.49% stronger F1-Score. This gap underscores the impact of V-SAM's tailored optimization, prompting, and skip-connection design, which DET-SAM lacks due to limited adaptation for dense medical tasks. A detailed comparison of all models is summarized in Table 8, and the corresponding performance trends are visualized on the right side of Figure 10.

**Figure 11:**
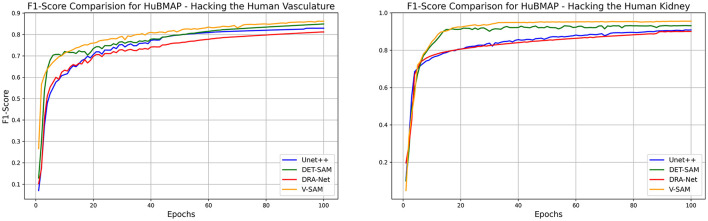
The variations in F1-Score per epoch by different model on both datasets from (left to right) for HubMAP-1 and HubMAP-2 respectively.

Table 9 further compares V-SAM with state-of-the-art (SOTA) models on the HubMAP-2 dataset. V-SAM achieves an Accuracy of 97.65% and an F1-Score of 95.54%, outperforming LinkNet (EfficientNet-B3 backbone) in F1-Score by 1.21%, DS-FNet (AU-Net backbone) by 0.36%, and SegNeXt (MSCAN-S backbone) by 0.08%. While LinkNet achieves the highest Accuracy (99.70%), its reliance on EfficientNet limits its ability to balance precision and recall, resulting in a lower F1-Score. This limitation stems from the inherent constraints of convolutional architectures, which struggle to model long-range dependencies and global context effectively. Similarly, DS-FNet and SegNeXt, despite their strong performance, are constrained by their convolutional architectures, which are less effective at capturing fine-grained details and global structures compared to transformer-based models like V-SAM. The F1-Score comparison among both datasets are given in Figure 11.

**Table 9:** Comparison of InFeNet with SOTA for HubMAP-2.

**Model**	**Publication / Year**	**Backbone**	**Accuracy (%)**	**F1- Score (%)**
EnsembleDLNet (33)	AJSE-22	ResNet-50	97.50	91.50
DS-FNet (35)	CMIG-22	AU-Net	—	95.05
UNet (48)	Soft Computing-23	EfficientNet-B4	99.68	90.60
LinkNet (38)	Digital Imaging-23	EfficientNet-B3	**99.70**	94.20
SegNeXt (40)	Neurocomputing-24	MSCAN-S	—	95.33
CNN-TransXNet (3)	IJCIS-24	TransXNet	85.13	82.80
V-SAM	—	ViT-B	97.65	**95.54**

The bold values showing best results.

In contrast, CNN-TransXNet (TransXNet backbone) performs poorly, with V-SAM achieving 12.52% higher Accuracy and 12.61% better F1-Score. This significant performance gap is likely due to inefficiencies in CNN-TransXNet's hybrid convolutional-transformer design, which fails to fully leverage the strengths of either architecture. V-SAM, on the other hand, excels due to its pure transformer-based architecture (ViT-B backbone), which is specifically designed to model global dependencies and intricate structures. Additionally, V-SAM's advanced fine-tuning techniques and optimization strategies ensure that it adapts effectively to the dataset, delivering precise and balanced results. These features solidify V-SAM's position as a leading model for medical image segmentation tasks, as demonstrated by its superior performance across multiple metrics. The comparison with SOTA models is provided in Table 9.

**Figure 12:**
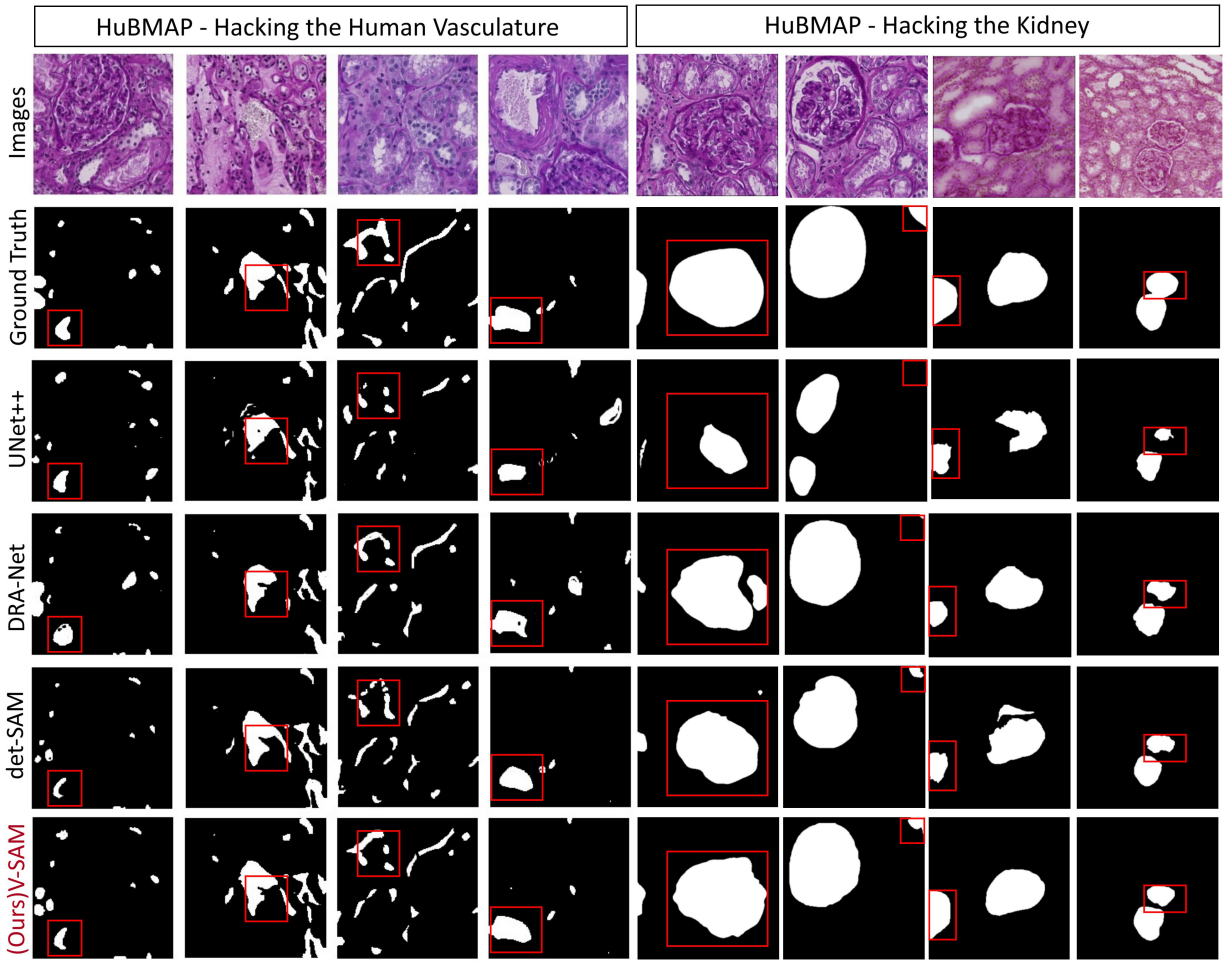
Visual comparison of the proposed V-SAM model with SOTA models on the HubMAP-1 and HubMAP-2. From left to right, the first four images represent HubMAP-1, while the following four images depict HubMAP-2.

The segmentation results provide further insight into the performance of each model when applied to kidney histology images (Figure 12). UNet++ captures the primary structures but struggles with accurately segmenting the finer regions. Over-segmentation is observed in areas such as the top-left and top-right corners, where boundary details are not preserved, and gaps in segmentation appear in some areas, as highlighted in the red boxes. DRA-Net improves upon these issues by showing better overall segmentation continuity, though boundary artifacts persist, and small structures remain inadequately segmented. Det-SAM performs moderately well, showing better refinement of major structures, but there is a tendency to under-segment, particularly in regions containing finer details or smaller clusters. In contrast, the V-SAM model excels by closely matching the ground truth, accurately capturing both large and small features with minimal errors. It achieves clean and precise boundaries, successfully preserving structural integrity even in complex regions. This superior performance is likely due to the model's ability to utilize spatial, channel, and temporal attention mechanisms that enhance feature extraction and refinement of segmentation masks. Additionally, V-SAM demonstrates better generalization, effectively handling variability in texture and shape across different regions of the images. Therefore, V-SAM maintains the optimal balance between segmentation accuracy and detail preservation, making it the best-performing model for this task.

## 6 Discussion

The experimental results demonstrate that the proposed V-SAM model achieves superior performance compared to several state-of-the-art (SOTA) segmentation models, including UNet++, nnUNet, DRA-Net, and DET-SAM, on both the HubMAP-1 and HubMAP-2 datasets. These improvements are largely attributed to V-SAM's transformer-based architecture, specifically the ViT-B backbone, which effectively captures both global contextual relationships and fine-grained structural details. Robust data augmentation and careful fine-tuning of the attention mechanism further enhance the model's ability to handle complex textures and morphological variations common in medical images. V-SAM achieved an accuracy of 89.31%, a Recall of 87.29%, and an F1-score of 86.17% on HubMAP-1, and an accuracy of 97.65%, Recall of 95.62%, and F1-score of 95.54% on the more challenging HubMAP-2 dataset. These results indicate consistent improvements across both coarse and fine structures, as further validated by visual comparisons showing V-SAM's ability to delineate both large anatomical regions and smaller entities like glomeruli critical for accurate diagnosis and treatment planning.

Although V-SAM leverages point-based prompts to guide segmentation, the inclusion of sparse point prompts is deliberate. This approach addresses limitations observed in purely automated models, particularly for tasks involving complex, low-contrast structures such as kidney glomeruli, where fully automated methods often struggle to achieve the necessary precision. These prompts enable precise localization and targeted correction, offering enhanced control and reliability in edge cases where automated models tend to fail. The integration of gradient-based refinement further allows sub-pixel accuracy with minimal computational cost, making the approach viable for semi-supervised clinical workflows. This trade-off balances automation with interpretability and expert oversight, which is often essential in real-world medical applications.

Despite its strong performance, V-SAM does come with certain limitations. The generalizability to other imaging modalities and anatomical regions remains an open area for exploration. Future work will focus on reducing dependence on manual prompts while retaining performance gains, potentially through active learning or self-supervised refinement mechanisms. Overall, V-SAM presents a promising advancement in medical image segmentation, offering a compelling balance between automation and precision, and setting the stage for more robust and adaptable diagnostic tools in computational pathology.

## 7 Conclusion

In this study, we introduced V-SAM, a novel and efficient architecture tailored for medical image segmentation, with a specific focus on glomerulus segmentation in kidney images. V-SAM integrates a promptable paradigm, a V-shaped structure, and skip connections to effectively capture fine-grained details and preserve critical spatial information. The addition of adapter layers facilitates efficient fine-tuning of the pre-trained SAM, while the point-based prompt mechanism enhances the model's ability to localize low-contrast and fragmented structures with greater accuracy. Moreover, the upsampling adapter improves segmentation results by recovering high-resolution details. Experimental evaluations on the HubMAP-1 and HubMAP-2 datasets highlight V-SAM's superior performance. On HubMAP-1, V-SAM outperforms DET-SAM with improvements of approximately 5.3%, 4.2%, and 6.2% in accuracy, Recall, and F1-score, respectively. On the more challenging HubMAP-2 dataset, V-SAM surpasses DET-SAM by about 4.0%, 4.6%, and 6.1% in accuracy, Recall, and F1-score, respectively. These results demonstrate V-SAM's ability to capture global context and long-range dependencies, offering a clear advantage over traditional convolutional backbones.

In conclusion, V-SAM demonstrates its potential as a robust and efficient solution for medical image segmentation, particularly for complex tasks such as segmentation of the glomerulus in kidney images. With its adaptability, computational efficiency, and superior performance, V-SAM is well suited for clinical applications. Future work will focus on expanding V-SAM to other medical imaging tasks and further refining its architecture to optimize both performance and real-time usability in clinical settings.

## Data Availability

Publicly available datasets were analyzed in this study. This data can be found here: https://www.kaggle.com/competitions/hubmap-kidney-segmentation; https://www.kaggle.com/competitions/hubmap-hacking-the-human-vasculature.
